# The Second Paragraph of *the Organon*

**Published:** 1892-05

**Authors:** 


### HOMŒOPATHIC PHYSICIAN,

A MONTHLY JOURNAL OF

HOMŒOPATHIC MATERIA MEDICA AND CLINICAL MEDICINE.

"If our school ever gives up the strict inductive method of Hahnemann, we are lost, and deserve only to be mentioned as a caricature in the history of medicine."—CONSTANTINE HERING.

Vol. XII. MAY, 1892. No. 5.

### EDITORIAL.

THE SECOND PARAGRAPH OF *The Organon*.—The editorial in the March number of this journal, it will be remembered, was a short commentary upon the first paragraph of *The Organon*.

We have now to make a few remarks upon the second paragraph, which reads thus:

"The perfection of a cure consists in restoring health in a prompt, mild, and permanent manner; in removing and annihilating disease by the shortest, safest, and most certain means, upon principles that are at once plain and intelligible."

How shall we proceed "in restoring health in a prompt, mild, and permanent manner"?

It cannot be done by giving anodynes for pain, because the effect of the anodyne is simply to remove consciousness and the power of expression of pain until the paroxysm shall have passed. Furthermore, the patient is put into a state of depression of his vitality that remains for hours and days afterward, and what is more does not exempt him from a renewal of the attacks of pain.

It cannot be done by giving massive doses of Quinine for an attack of ague, because a dose of Quinine under such circumstances while it undoubtedly will cause the chill to disappear in most instances, nevertheless only suppresses its manifestation.

Meanwhile the energy that caused the chill is bottled up, to break out again at some future period, when the doses must be larger to accomplish the suppression. The time comes at last when no amount of Quinine will "break" the chill, and so the patient is not restored to health in either a prompt, a mild, or a permanent manner.

A close consideration of the subject will convince the inquirer that these methods must be abandoned if he would succeed in restoring health according to the terms demanded by the text.

It will be found on further consideration that it is not possible to know anything about a given drug or what its relationship to the animal economy may be, until it is first tested or "proved" upon the healthy. Here then is the first step in Homœopathy. The testing of medicines upon healthy individuals unfolds to our view upon what organ or organs a given drug acts, and under what circumstances or conditions it produces its effects.

The administration of medicines according to the law of similars is not the absurdity it appears to be when we look beneath the surface. The symptoms of a disease enable us to locate that disease. If we would break its power for evil we must bring about a meeting between it and some drug.

We cannot tell what is the mode of action of the drug upon the disease energy under such circumstances, but it is certain that if we can produce a collision between them, the one will annihilate the other.

How shall we bring about this collision? The answer is by giving a drug whose symptoms, when it is tried upon the healthy, shall closely correspond with those of the disease, by which we are assured that the drug locates itself in the immediate vicinity of the disease, and under the same conditions. The symptoms then are the guides or finger-boards, by which we shall accomplish this end. This surely is not "symptom covering," as is so commonly asserted. The *real* symptom coverer, as has been before remarked in this journal, is he who disguises pain with an opiate; suppresses a chill with Quinine; combats constipation with a purgative; suppresses diarrhœa with opiates and the chalk mixture, and uses other means on the same plan.

We prove medicines upon the healthy, therefore, because that method will alone give us a view of the effect of any particular drug upon the hnman organism and judge of its sphere of action in disease conditions.

We prescribe medicines according to the law of the similars, because it is only by giving a medicine whose symptoms agree with those of the patient, that we are assured that we are locating a resisting energy in the neighborhood of the morbific power which shall prevent its further progress.

To nine out of ten of the readers of this journal, the principles of Homœopathy are so familiar that the foregoing remarks will seem trite and commonplace. To all such it may be said that this article is not intended for their perusal, since most of them, it is well known, may express these thoughts in much better terms. Many of them *have* done so, as their articles which grace these pages from time to time will testify. But there is a class of younger minds growing up in the profession to whom these principles are not so familiar; who stand in need of this teaching. They are crowding forward, and swelling the subscription list of THE HOMŒOPATHIC PHYSICIAN, and so the same teaching of our principles and practice must be repeated over and over again if we would succeed in printing it indelibly upon their minds, and make them so familiar with it that they will come to practice it in its purity.

